# Factors that impact burnout and psychological wellbeing in Australian postgraduate medical trainees: a systematic review protocol

**DOI:** 10.1186/s13643-021-01809-z

**Published:** 2021-09-24

**Authors:** Belinda Balhatchet, Heike Schütze, Nicole Williams, Bruce Ashford

**Affiliations:** 1 University of Wollongong, Northfields Avenue, Wollongong, 2522 Australia; 2 Australian Orthopaedic Association, Level 26, 201 Kent St, Sydney, NSW 2000 Australia; 3 Australian National University, ACT, Canberra, 0200 Australia; 4 UNSW Sydney, Sydney, NSW 2052 Australia; 5 University of Adelaide, North Terrace, Adelaide, SA 5005 Australia; 6 Women & Children’s Hospital, 72 King William Rd, North Adelaide, SA 5006 Australia; 7 Illawarra Shoalhaven Local Health District, Warrawong, NSW 2505 Australia

**Keywords:** Burnout, Wellbeing, Mental health, Quality of life, Australia, Postgraduate, Medical, Health, Education, Trainees

## Abstract

**Background:**

The stressful nature of medical training and other work-related factors put postgraduate medical trainees at high risk of burnout and poor psychological wellbeing. This has negative implications for patient care and the effectiveness of the healthcare system. The structure of the healthcare system and postgraduate medical education in Australia is different to that of other countries. Whilst a significant body of research exists on burnout and wellbeing in trainees in the USA, evidence specific to Australian trainees is lacking. The aim of this review is to synthesise the current knowledge on the factors that impact burnout and psychological wellbeing in Australian postgraduate medical trainees.

**Methods/design:**

A systematic review will be conducted across eight digital databases: Academic Search Complete, MEDLINE, Embase, Web of Science*,* PsychInfo*,* Scopus*,* CINAHL Plus and Informit Health Collection. Peer reviewed empirical studies and relevant grey literature published after 2000 that address an aspect of burnout or psychological wellbeing in Australian postgraduate medical trainees will be included. Two reviewers will independently review each article against the inclusion and exclusion criteria, with disagreements resolved via discussion and consensus. Data will be extracted using a standard form and quality will be assessed using the assessment tools available from the Joanna Briggs Institute. A thematic narrative synthesis of the studies will be presented, along with an assessment of current gaps in the literature and areas for future research.

**Discussion:**

This review will be the first to integrate the evidence on burnout and psychological wellbeing specific to Australian postgraduate medical trainees. The findings will contribute to a better understanding of the factors that impact burnout and psychological wellbeing in this population and will lay the foundation for future research into appropriate strategic interventions.

**Systematic review registration:**

This protocol has been registered in the International Prospective Register of Systematic Reviews (PROSPERO: CRD42020203195).

**Supplementary Information:**

The online version contains supplementary material available at 10.1186/s13643-021-01809-z.

## Background

The pathway to becoming a medical specialist in the Australian healthcare system is long and arduous. Trainees must complete at least four years of university education followed by internship and several years of postgraduate training in their specialty of choice [1]. Consistent growth in medical graduate numbers has led to increased competition for specialist training places and reduced employment opportunities for new fellows across many specialty training programs [2]. In some specialties, less than half of those who apply for selection are successful in gaining a post [3]. Many trainees make significant personal sacrifices in order to improve their chances of succeeding in the selection process and training program for their chosen specialty [4].

Burnout is a prolonged response to ongoing stressors and is characterised by three key dimensions: emotional exhaustion, depersonalisation and reduced sense of personal achievement [5]. Recent research has shown that burnout symptoms amongst junior doctors and specialist trainees are common [6, 7] and trainees are more susceptible to poor mental health and suicide than the general population [8–10]. Work-related factors have a greater impact on trainee burnout and psychological wellbeing than non-work-related factors [11]. Potential contributing factors include lack of work-life balance, exposure to death and injury, experiences of discrimination and harassment, lack of support networks, and long work hours [12–17].

Poor trainee wellbeing negatively impacts patient care by affecting the quality of patient discussions, contributing to depersonalisation in patient interactions and increasing rates of medication errors [18, 19]. Physician burnout also has consequences for productivity and healthcare reform [20, 21]. Despite this, it can be difficult to identify and address poor psychological wellbeing due to trainees’ reluctance to seek support for fear of appearing weak or clinically incompetent [22]. It has been suggested that this attitude is transmitted to new trainees via the socialisation process of the ‘hidden curriculum’: the cultural perpetuation of hazardous behaviours between doctors which undermines the messages of the prescribed teaching curriculum [23, 24]. Whilst various interventions have been trialled in an effort to address the problem, including mentoring programs, wellbeing and mindfulness workshops, stress management training, exercise programs and psychotherapeutic techniques [25–30], there have been mixed results across the various domains of wellbeing.

A number of recent systematic reviews and meta analyses have been conducted on medical trainee wellbeing [15, 19, 31–34]; however, we are not aware of any work specifically on Australian trainees. Studies of Australian trainees have been included in previous reviews, but have not been assessed in isolation from studies of populations in other countries. As the structure of the healthcare system and postgraduate medical education in Australia is different to that of other countries, it is important to assess the evidence regarding burnout and psychological wellbeing in Australian trainees in isolation before attempting to design and implement solutions. The aim of this review is therefore to identify the factors that impact burnout and psychological wellbeing in Australian postgraduate medical trainees.

## Methods/design

This systematic review protocol has been reported according to the Preferred Reporting Items for Systematic Reviews and Meta-Analyses 2015 checklist (PRISMA-P) [35] (Additional File 1). Due to the diverse types of qualitative and quantitative studies and the heterogeneity of the sample that will be included in the review, meta-analysis will not be possible. The results will be synthesised thematically. The protocol was registered in advance with the International Prospective Register of Systematic Reviews (PROSPERO): CRD42020203195.

### Review questions

The overarching research question for this systematic review is:

1. What impacts burnout and psychological wellbeing in Australian postgraduate medical trainees?

### Search strategy

This systematic review will aim to include empirical peer-reviewed studies published in academic journals. First, the lead author (BB) will undertake a structured search in the following databases:

- Academic Search Complete
- MEDLINE full text
- Embase
- Web of Science
- PsychInfo
- Scopus
- CINAHL Plus
- Informit Health Collection

These databases have been chosen as they provide access to current, scholarly peer-reviewed journals in the fields of health, psychology and education. Search terms will be set up using the PIO (Population, Interest, Outcome) framework. Initial search terms are outlined in Additional File 2.

The initial search will be re-run immediately before analysis commences and any additional studies retrieved for inclusion. The database searches will be restricted to include articles published from 1 January 2000 onwards. Only articles available in English will be included. A search of the reference lists of included studies will also be conducted to identify additional articles for inclusion. A Google search will be run to identify any relevant grey literature for inclusion.

### Inclusion and exclusion criteria

#### Participants

For the purposes of this review, postgraduate medical trainees will be defined as graduates with a medical degree who are in working in pre-vocational (intern, resident) or vocational (registrar) positions in the Australian healthcare system. Studies focused on medical students, consultants and senior doctors will be excluded, including those where results for postgraduate trainees are not reported separately from another population. Studies investigating Australian and New Zealand trainees together will be included, due to the similarity of training programs and the trans-Tasman governance of many medical colleges. Due to the scarcity of literature available, no limits will be applied in terms of age, gender, ethnicity or specialty training program.

#### Exposures

For this review, the exposures of interest are defined as any factor of the work environment that demonstrates an association with, or an impact on, an aspect of psychological wellbeing. Examples of factors may include, but are not limited to, assessments and examination, working hours, support networks, experiences of discrimination or harassment, work-life balance and exposure to death and injury. Associations may be positive or negative.

#### Outcomes

The primary outcomes for this review will be any measure of one or more aspects of psychological wellbeing in Australian postgraduate medical trainees. Aspects of psychological wellbeing include, but are not limited to, burnout, mental health (including depression and anxiety), quality of life, and stress. Outcome data may have been collected by any qualitative or quantitative data collection method, or a combination thereof. Studies that focus solely on physical wellbeing rather than psychological wellbeing will be excluded.

#### Types of studies

This review will include published empirical peer-reviewed studies. All quantitative, qualitative and mixed-methods study types will be considered eligible for inclusion. Review papers, study protocols, commentaries, opinion pieces, letters to the editor, magazine articles and discussion papers will be excluded. Grey literature will be eligible for inclusion. Abstracts will not be included, but efforts will be made to locate an associated full-text publication. In order to include the most up-to-date literature, the search will be limited to articles published from 1 January 2000 onwards.

### Study selection

Search results will be downloaded to Microsoft Excel for review. Duplicates will be manually removed. Several steps will be undertaken to ensure rigour. Firstly, two screening processes will take place. In the first, titles and abstracts of each study will be downloaded and assessed by a minimum of two reviewers against the inclusion and exclusion criteria, and any studies not meeting the inclusion criteria will be excluded. Any disagreements in relation to inclusion or exclusion of studies will be discussed between the reviewers and agreed by consensus. If consensus cannot be reached, a third reviewer will be invited.

In the second screening, the full-text versions of the remaining articles will be independently reviewed by at least two reviewers. Disagreements will be discussed amongst the reviewers and agreed by consensus. If consensus cannot be reached, a third reviewer will be invited. Reasons for exclusion will be documented at each stage. If there is insufficient information to determine eligibility, the authors of the study will be contacted for further clarification. If further information is not available, the study will be excluded.

A detailed PRISMA flowchart of the selection process will be included in the final review.

### Data extraction

Data extraction will be conducted for all eligible articles by a member of the research team, based on a modified version of the standardised data extraction form developed by Hall et al. [36]. Extracted information will include the following: title, author, publication date, aims, study type, data collection tools, population, inclusion and exclusion criteria, response rate, recruitment method, limitations, statistical analysis, results and conclusions. Data extraction for all articles will be reviewed by a second author to ensure accuracy and reliability. It is expected that the data extraction will be an iterative process whereby the data extraction template may be modified as the process unfolds.

### Confidence in cumulative evidence

Confidence in the findings of this review will be assessed using the Confidence in the Evidence from Reviews of Qualitative research (CERQual) approach [37]. This will provide an assessment of the extent to which the findings are a reasonable representation of the phenomenon of interest (in this case, factors that impact burnout and psychological wellbeing in Australian postgraduate medical trainees). In essence, it provides an evaluation of the level of confidence that can be placed in the findings of this review.

The evaluation will provide an assessment of the following:

1. Methodological limitations: An evaluation of any problems in the design or conduct of the primary studies, guided by the quality assessment process described below;
2. Relevance: An assessment of the extent to which the primary studies are applicable to the context and aims of the review;
3. Coherence: An assessment of the extent to which the tendencies, patterns and relationships identified in the findings are grounded in data from the primary studies;
4. Adequacy of data: An overall assessment of the evidence supporting the review finding.

### Quality assessment

Each article will be assessed for quality and potential bias using the critical appraisal tools available from the Joanna Briggs Institute [38]. These checklists use a three-option grading system of: include, exclude and seek further information based on desirable and undesirable effects, quality of evidence, values and preferences and costs [38]. This suite was chosen as it contains 13 checklists for different study types, thereby providing a consistent assessment tool across all study types. For qualitative research in particular, the JBI tool scores higher for dependability and credibility compared to other similar tools [39]. The most appropriate tool will be selected for each study type. Quality assessment will be conducted by two reviewers and disagreements resolved by discussion and consensus.

### Analysis and reporting

Data will be analysed using a convergent qualitative synthesis process [40]. Study results (quantitative, qualitative and mixed methods) will be transformed into qualitative findings such as themes, concepts and patterns [40]. The thematic analysis process will be undertaken using a hybrid deductive-inductive approach, where data is initially assigned to predefined themes (deductive) and themes are revised or created based on data (inductive; [40]). Rigour in the transformation and analysis process will be achieved by discussion and consensus between three authors, with the fourth author providing arbitration for any disagreements [40]. Narrative synthesis will then be used to relate these findings to the research question. An appraisal of the quality of the included studies will be provided based on the quality assessment process. Gaps in the literature and areas for future research will be identified.

## Discussion

There are an increasing number of studies on burnout and psychological wellbeing in medical trainees and junior doctors, but there are currently no systematic reviews that are specific to the Australian setting. This protocol describes the planned methodology for a systematic review focused on burnout and psychological wellbeing in Australian postgraduate medical trainees. The review will explore the scope of the problem specific to the Australian training landscape and will lay the foundation for future research into appropriate strategic interventions.

A limitation of this review is the exclusion of studies that are not in English and the publication restriction from 2000 onwards. The time restriction was applied in order to restrict the final list of studies to a manageable number and to ensure relevance of the findings. We expect that the review may present challenges in the heterogeneity in methods for defining and measuring psychological wellbeing, as well as in study settings, which may limit interpretability of results. We also anticipate that some studies will be ‘borderline’ in their relevance to trainee wellbeing, which we will attempt to mitigate by discussing these studies amongst multiple reviewers and coming to a consensus decision on inclusion or exclusion.

Findings from this review will be disseminated in peer-reviewed publications, conference presentations and as part of a doctoral thesis. The review will be of value to policy-makers, health services and medical training institutions who are responsible for the wellbeing of the next generation of Australian medical specialists.

## Supplementary Information

**Additional file 1.** PRISMA-P 2015 Checklist.

**Additional file 2.** Search terms.

## Data Availability

The datasets used and/or analysed during the current study are available from the corresponding author on reasonable request.

## Abbreviations

CINAHL: Cumulative Index to Nursing and Allied Health Literature database
MEDLINE: Medical Literature Analysis and Retrieval System Online
EMBASE: Excerpta Medica Database
PIO: Population, Interest, Outcome framework
PRISMA: Preferred Reporting Items for Systematic Reviews and Meta-Analyses
PROSPERO: International Prospective Register of Systematic Reviews
