# Supplementary material for: Factors that impact burnout and psychological wellbeing in Australian postgraduate medical trainees: a systematic review protocol
Source: Syst Rev. 2021 Sep 24;10:257. doi: 10.1186/s13643-021-01809-z (PMC8464131; doi:10.1186/s13643-021-01809-z)
Supplement: Supplementary file 2 — Additional file 2. Search terms. [file 13643_2021_1809_MOESM2_ESM.docx]

**Additional File 2: Search terms**

| Population | Australia* AND (Trainee* OR registrar* OR junior doctor*) |
| --- | --- |
| AND | |
| Outcome | well?being OR mental health OR “quality of life” OR burnout OR depression OR anxiety |
